# Structure–Performance Correlation Inspired Platinum-Assisted Anode with a Homogeneous Ionomer Layer for Proton Exchange Membrane Water Electrolysis

**DOI:** 10.3390/polym16020237

**Published:** 2024-01-15

**Authors:** Feng Cheng, Tian Tian, Rui Wang, Hao Zhang, Liyan Zhu, Haolin Tang

**Affiliations:** 1 National Energy Key Laboratory for New Hydrogen-Ammonia Energy Technologies, Foshan Xianhu Laboratory, Foshan 528200, China; 2 Wuhan Institute of Hydrogen and Fuel Cell Industrial Technology, 555 Cultural Avenue, Hongshan District, Wuhan 430070, China; 3 State Key Laboratory of Advanced Technology for Materials Synthesis and Processing, Wuhan University of Technology, Wuhan 430070, China

**Keywords:** PEMWE, anode, ionomer layer, Ir oxide, Platinum

## Abstract

PEMWE is becoming one of the most promising technologies for efficient and green hydrogen production, while the anode OER process is deeply restricted by the now commercially used iridium oxide with sluggish reaction kinetics and super high cost. Deeply exploring the essential relationship between the underlying substrate materials and the performance of PEMWE cells while simultaneously excavating new practical and convenient methods to reduce costs and increase efficiency is full of challenges. Herein, two representative kinds of iridium oxide were studied, and their performance difference in PEMWE was precisely analyzed with electrochemical techniques and physical characterization and further linked to the ionomer/catalyst compound features. A novel anode with a uniform thin ionomer coating was successfully constructed, which simultaneously optimized the ionomer/catalyst aggregates as well as electrical conductivity, resulting in significantly enhanced PEMWE performance. This rigorous qualitative analysis of the structure–performance relationship as well as effective ionomer-affinitive optimization strategies are of great significance to the development of next-generation high-performance PEM water electrolyzers.

## 1. Introduction

Green hydrogen is one of the most potential alternatives to traditional fossil energy sources for achieving carbon neutrality [1,2,3]. The development of hydrogen energy technology is closely linked to macro-level industrial chain planning, yet it is profoundly contingent on the innovations of frontier key materials [4,5,6,7,8]. Iridium (Ir)-based nanomaterials have been recognized as the most efficient oxygen evolution reaction (OER) catalysts and are widely investigated in proton exchange membrane water electrolysis (PEMWE) equipment [9,10,11,12]. However, due to the high cost of Ir and the large space of overpotential improvement toward the OER, a lot of research has been completed to reveal the optimum process conditions for membrane electrode assembly (MEA) [13,14], the transformation of the active site for Ir species [15], the degradation mechanism of the Ir oxide-based PEMWE cell [16,17], etc. Lyu et al. [18] systemically evaluated the effect of IrO_2_ ink aging on the catalyst layer structure and PEMWE performance. Only a slight increase in particle size and viscosity of the ink appeared during a time period of 14 days, and there was less effect on the structure of the catalyst/ionomer interface and catalyst layer. Song et al. [19] investigated the effect of Nafion content on the performance of a PEMWE single cell at 80 °C and ambient pressure; the best content of 7.0% Nafion was obtained with the lowest value of anode charge transfer resistance. Xu et al. [20] demonstrated an IrO*_x_*·*n*H_2_O catalyst combining crystalline and amorphous IrO_2_ with excellent performance, which benefitted from the lattice water sustainably participating in oxygen exchange. Shi et al. [21] reported a one-dimensional Ir oxide nanorod supported on Sb-doped SnO_2_ with extremely high activity toward the OER. It was concluded that the interface of IrO_2_ and SnO_2_ performed a lower degree of Ir oxidation and promoted the facile adsorption of oxygenated species. The only issue is that this positive effect has not been confirmed with testing on a single cell, since PEMWE performance based on the MEA is not only related to the catalytic activity of Ir oxides in the anode. Although a large amount of research has been conducted [22,23,24], a further in-depth analysis of the inner relationship between structural properties and electrolytic performance of anode, which could further promote the development of new technologies for performance promotion, is of extremely high value for basic research on key materials and the commercialization process of water electrolysis. The anode catalyst design with other functional components is one of the most famous methods for activity regulation. Among these, Pt has been frequently used in alloying, electronic structure modifications, morphology regulation, etc. Ticianelli et al. synthesized Pt/IrO_2_ using a post-loading process of Pt. Catalysts with various Pt: Ir ratios performed differently under three aging protocols, and the Pt/IrO_2_ 1:9 material exhibited better activity balance between the OER and ORR (oxygen reduction reaction) [25]. Yang et al. modified the electronic structure of IrO_2_ with the electrodeposition of Pt. The corresponding OER and HER activities were improved, and two-electrode electrolyzers were simultaneously assembled, which exhibited attractive performance under universal conditions and showed certain potential in practical applications [26]. However, these research studies were all goal-oriented with performance improvement at the electrode or liquid electrolyzer level using relatively complex processes. In other words, the necessary condition for the intrinsic activity was taken into account, but the inner structure of the catalyst layer, which seriously affects mass transfer, and the true expression of hydrolysis were not explored in depth. The adsorption of the ionomer sulfonic group has been suggested to poison the Pt and Ir active sites, further compressing the ORR and OER kinetics at both the microelectrode and MEA levels [27]. The ionomer content reported in the literature fluctuates widely, which directly affects the anode charge transfer resistance [19,28,29]. It was shown that the ionomer plays an important role in interacting with Ir oxide particles on the mass transfer in the aggregates, while research on the catalyst–ionomer structure is meaningful to the optimization of new and efficient MEA for water electrolysis [30]. The catalyst ink and acquired catalyst layer impact PEMWE performance significantly. Therefore, developing new materials and technology considering the relationship between structure features and cell performance is of great importance for high-performance green water electrolysis.

Thus, in this study, two representative candidates, IrO*_x_*·*n*H_2_O and IrO_2_ with significantly different crystal structure features, are selected to investigate the origin of performance differences in PEMWE between them. The inflection point of performance differences occurs at the current density of around 300 mA/cm^2^, which demonstrates the complexity of factors affecting electrolytic cell performance. Therefore, a complete set of electrochemical measurements including linear sweep voltammetry (LSV), electrochemical impedance spectroscopy (EIS), cyclic voltammetry (CV), and a series of reasonable characterization methods such as X-ray photoelectron spectroscopy (XPS), scanning electron microscopy (SEM), and small angle X-ray scattering (SAXS) are applied to research the intrinsic activity and structural features of the catalyst, ink, and catalyst layer. Then, the factors that cause the performance difference in the catalyst in the electrolytic cell are found, and based on this discovery, a novel anode with low-iridium and ionomer-affinitive structure is constructed, which greatly improves the water electrolysis performance of IrO*_x_*·*n*H_2_O. The research methods for identifying the origin of performance differences in various catalysts in electrolysis single cells and the new construction methods for the high-performance anode are of great significance for the application in devices of other electrocatalysts.

## 2. Experimental Section

Chemicals: IrO*_x_*·*n*H_2_O and IrO_2_ were purchased from Shaanxi Kaida chemical engineering Co., Ltd (Shaanxi, China). Pt/C (50 wt.%) was obtained from Tanaka Kikinzoku Kogyo (Tokyo, Japan). Pt black nanoparticles were purchased from Premetek Co. (Cherry Hill, NJ, USA). The 5 wt.% Nafion solution and Nafion 115 membrane were purchased from DuPont. All chemicals were used as received. Deionized water (18.25 MΩ/cm) was used.

Physical characterization: The morphology of the catalyst was analyzed with high-resolution transmission electron microscopy (HRTEM, Talos F200S, Thermo Fisher Scientific, Waltham, MA, USA) and energy-dispersive X-ray spectroscopy (EDS, Bruker, Germany). The 3D tomography of the catalyst was investigated with high-angle annular dark field scanning transmission electron microscopy (HAADF-STEM, FEI Tecnai G2 F30, Hillsboro, OR, USA, 300 kV). The XRD pattern was recorded on a RU-200B X-ray diffractometer. The electrical conductivity of the catalysts was measured using a semiconductor-powder resistivity instrument (ST-2722, Suzhou Jingge Electronic Co., Ltd., Suzhou, China). XPS spectra were recorded with X-ray photoelectron spectroscopy (AXIS SUPRA, Kratos Analytical, Manchester, UK). The Zeta potential and dispersion of particle size were performed using a NanoBrook 90Plus PALS instrument (Brookhaven, Holtsville, NY, USA). SEM images were acquired with a JSM-IT800 field emission scanning electron microscope. The microstructure of the catalyst layer was analyzed with a Xeuss 3.0 SAXS/WAXS system.

Electrochemical characterization: The electrochemical properties of the IrO*_x_*·*n*H_2_O and IrO_2_ catalysts were investigated in a classic three-electrode system using a CHI660E electrochemical workstation [31]. A glassy carbon electrode (d = 5 mm) was used as the working electrode, and a carbon rod and reversible hydrogen electrode (RHE) were used as the counter electrode and the reference electrode, respectively. All the measurements were conducted in a freshly configured 0.1 M HClO_4_ solution. LSV curves were recorded at a rotating speed of 1600 rpm. EIS was carried out at 1.5 V vs. RHE with an amplitude of 10 mV within the frequency of 100,000 Hz to 0.01 Hz. CV was recorded at a potential range of 0–1.2 V vs. RHE with a scan rate of 20 mV/s. For measurement, the catalyst powder was ultrasonically dispersed in media containing water, isopropyl alcohol, and 5 wt.% Nafion solution (the mass ratio of catalyst: water: isopropyl alcohol: Nafion was 5 mg: 0.9 mL: 0.1 mL: 20 μL). Then, 5 uL of catalyst ink was drop-cast on the glassy carbon electrode with a catalyst loading of 0.127 mg/cm^2^.

MEA preparation: MEAs were produced with a conventional Decal transfer method [32]. Pt/C and Nafion 115 were used as the cathode catalyst and membrane. Pt nanoparticles, IrO*_x_*·*n*H_2_O, IrO_2_, Pt/IrO*_x_*·*n*H_2_O-1, and Pt/IrO*_x_*·*n*H_2_O-2 were used as the anode catalysts. The catalyst layers were fabricated from the catalyst ink with balling, sonication, blade casting, and the heat press transfer process successively. The active area of MEAs was 25 cm^2^. The catalyst loading (Pt) in the cathode was around 0.4 mg/cm^2^. The catalyst loading in the anode was 1.2 mg/cm^2^, and the mass ratio of Pt: IrO*_x_*·*n*H_2_O in the catalyst layer of Pt/IrO*_x_*·*n*H_2_O-1 and Pt/IrO*_x_*·*n*H_2_O-2 was 4:1 and 3:1.

Single-cell test: All the single-cell performances of the MEAs were investigated using an IPS PTC-05100E large current electrochemical workstation [32]. Deionized water was used as a reactant. The test temperature was 60 °C, and the feed rate of water was 10 mL/min. The MEAs were activated by applying the voltage load until the current reached a steady state. The I-V curves were recorded at various voltages for 5 min. High-frequency resistance (HFR) was recorded at 1000 Hz during the measurement.

## 3. Results and Discussion

**Figure 1:**
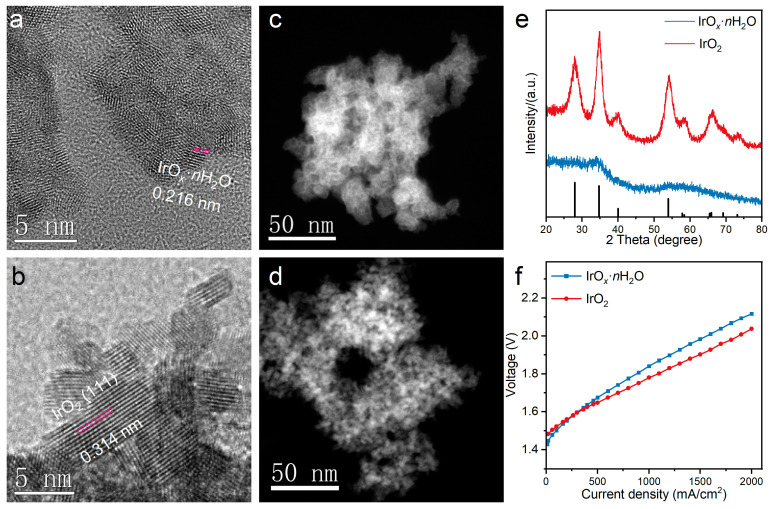
High-resolution TEM images of (**a**) IrO*_x_*·*n*H_2_O and (**b**) IrO_2_; HAADF-STEM images of (**c**) IrO*_x_*·*n*H_2_O and (**d**) IrO_2_; (**e**) XRD patterns of the IrO*_x_*·*n*H_2_O and IrO_2_; and (**f**) the I-V curves of single-cell PEMWE for IrO*_x_*·*n*H_2_O and IrO_2_.

The basic morphological and structural characteristics of IrO*_x_*·*n*H_2_O and IrO_2_ were investigated, and the corresponding images and patterns were recorded with HR-TEM, HAADF-STEM, and XRD equipment. As shown in Figure 1a, short and messy lattice stripes for IrO*_x_*·*n*H_2_O were observed, presenting dense and aggregated particle structures consisting of short-ordered nanoclusters (Figure S1) [20]. While, as shown in Figure 1b, a clear and ordered crystal lattice was observed, demonstrating the high crystallinity of IrO_2_ [33]. The *d*-spacing of the crystal marked in red was determined to be 0.314 nm using Digital Micrograph analysis, which corresponded to the (110) plane of IrO_2_. HAADF-STEM was also used to characterize the 3D tomography of the catalyst, as shown in Figure 1c,d. The structure of IrO*_x_*·*n*H_2_O was more continuous and had fewer voids, while IrO_2_ was loose and porous.

In addition, the XRD patterns of IrO*_x_*·*n*H_2_O and IrO_2_ were plotted, as shown in Figure 1e. There was only one broad peak located around 34.9 for IrO*_x_*·*n*H_2_O, which suggested a small grain size or amorphous characteristics and was consistent with the HRTEM result [20]. In contrast, there were seven sharp peaks at 28.0, 34.9, 40.1, 54.1, 58.2, and 73.2 in IrO_2_, corresponding to the (110), (101), (200), (211), (220), and (202) planes of IrO_2_ (PDF# 01-088-0288), which further confirmed it is highly crystalline. Considering the obvious differences in the structural properties of the two catalysts, the cell performance was demonstrated in the commercial PEM electrolyzer. As shown in Figure 1f, the current density of both catalysts increased with the rise in the voltage load. The voltages for IrO*_x_*·*n*H_2_O and IrO_2_ were 1.841 V@1000 mA/cm^2^, 2.117 V@2000 mA/cm^2^, and 1.781 V@1000 mA/cm^2^, 2.038 V@2000 mA/cm^2^. At a current density below 300 mA/cm^2^, the performance of IrO*_x_*·*n*H_2_O was better, while when the current was above 300 mA/cm^2^, IrO_2_ performed better. This could suggest that IrO*_x_*·*n*H_2_O had higher electrochemical activity compared with IrO_2_ and that the large voltage loss for IrO*_x_*·*n*H_2_O at high current density was mainly caused by hindered mass transport as well as higher ohmic polarization, which was induced by the catalyst/ionomer structural differences in the catalyst layer and even the distinctiveness of the catalysts themselves [34].

**Figure 2:**
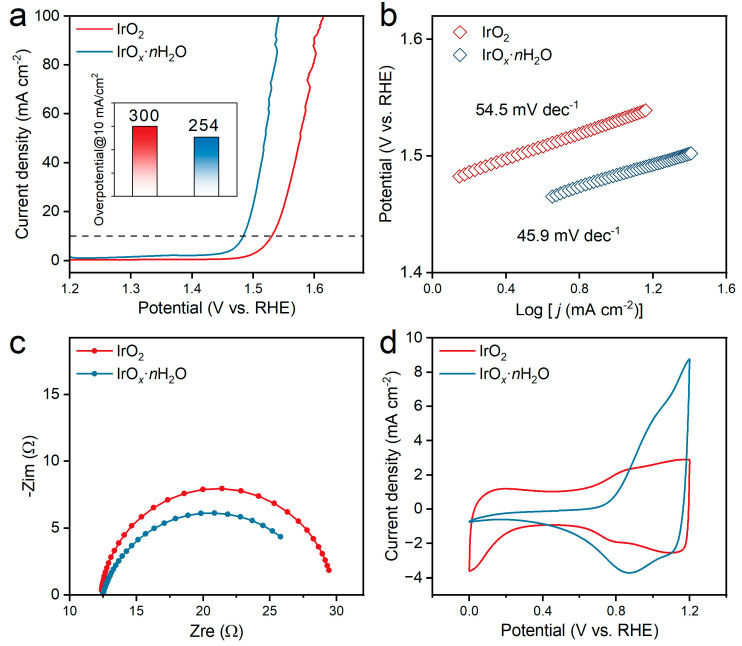
OER electrocatalytic performance of the studied catalysts in 0.1 M HClO_4_. (**a**) LSV curves of IrO*_x_*·*n*H_2_O and IrO_2_ (inset is the comparison of overpotential at 10 mA/cm^2^), (**b**) Tafel plots, (**c**) Nyquist plots of EIS, and (**d**) cyclic voltammetry curves of IrO*_x_*·*n*H_2_O and IrO_2_.

Therefore, it is very important to conduct a comparative analysis of the intrinsic activity of these two catalysts. The OER electrocatalytic performance of the catalysts in 0.1 M HClO_4_ was characterized using the rotating disk electrode (RDE), as shown in Figure 2. All the curves were recorded at a stable condition of the electrode after moderate activation [35]. The LSV curves of IrO*_x_*·*n*H_2_O and IrO_2_ are shown in Figure 2a. IrO*_x_*·*n*H_2_O performed better in OER activity than IrO_2_ with lower overpotentials at all current densities. Furthermore, the overpotential of IrO*_x_*·*n*H_2_O (254 mV vs. RHE) at 10 mA/cm^2^ was much lower than that of IrO_2_ (300 mV vs. RHE). Tafel plots are one of the most efficient methods to evaluate the mechanism and activity of material in electrocatalysis by acquiring the plot parameters of the exchange current density (reversible potential range of the OER) and Tafel slope [36,37]. The Tafel slope of IrO*_x_*·*n*H_2_O and IrO_2_ at low current density intervals were calculated as 45.9 and 54.5 mV/dec, respectively. The lower Tafel slope of IrO*_x_*·*n*H_2_O suggested that it had faster reaction kinetics in the rate-determining step in the OER [38,39].

Furthermore, EIS plots were carried out to investigate the charge transfer process in the OER. As shown in Figure 2c, the R_s_ value (the first intersection point of the data with the *x*-axis) indicated that the contact resistance and electrolyte resistance were the same with a value of about 12.5 ohm [40]. The resistance of the charge transfer (R_ct_) at the catalyst interface for IrO*_x_*·*n*H_2_O was about 15.0 ohm compared with 17.5 ohm for IrO_2_, revealing the fast charge transfer of IrO*_x_*·*n*H_2_O during the OER process [9]. The CV curves of IrO*_x_*·*n*H_2_O and IrO_2_ were also measured, as shown in Figure 2d. It was found that the CVs of IrO*_x_*·*n*H_2_O and IrO_2_ were similar, both displaying a typical electrochemical response with pseudocapacitive features. There were two pairs of reversible peaks located around 0.79 and 1.03 V vs. RHE, which corresponded to Ir(Ⅱ)/Ir(Ⅲ) and Ir(Ⅲ)/Ir(Ⅳ), respectively [41]. However, IrO_2_ exhibited more obvious characteristics of an electric double layer in the potential range between 0 and 1.2 V. This could be related to the differences in crystal structure and pore features between IrO*_x_*·*n*H_2_O and IrO_2_, which were already characterized with TEM and XRD. Overall, standard three-electrode measurements were carried out to investigate the electrocatalytic performance of IrO*_x_*·*n*H_2_O and IrO_2_, and IrO*_x_*·*n*H_2_O performed better with a lower overpotential of 46 mV than IrO_2_ and was confirmed to have higher intrinsic activity toward the OER. This was consistent with the analysis result for the cell performance of the PEM water electrolyzer.

**Figure 3:**
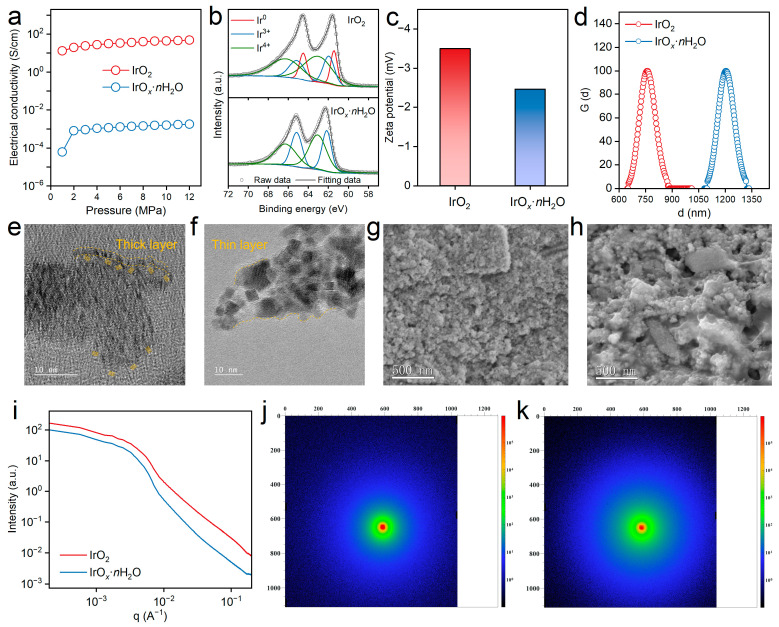
(**a**) The electrical conductivity of IrO*_x_*·*n*H_2_O and IrO_2_ as a function of pressure; (**b**) detailed Ir 4f spectra of IrO*_x_*·*n*H_2_O and IrO_2_; (**c**) Zeta potential and (**d**) size distribution curves of IrO*_x_*·*n*H_2_O and IrO_2_; high-resolution TEM images of the catalyst/ionomer structure of (**e**) IrO*_x_*·*n*H_2_O and (**f**) IrO_2_; SEM images of the catalyst layer of (**g**) IrO*_x_*·*n*H_2_O and (**h**) IrO_2_; (**i**) 1D SAXS profiles of the anode catalyst layer of IrO*_x_*·*n*H_2_O and IrO_2_; and 2D SAXS patterns of (**j**) IrO*_x_*·*n*H_2_O and (**k**) IrO_2_.

Moreover, an innovative and rational design of the catalyst layer, especially the anode, is essential for the development of high-performance PEM water electrolysis [42,43]. Under high current density, the electrochemical reaction rate is extremely fast, which faces the pressure of high-throughput mass transport and high-speed electron transfer [44]. Therefore, the state of the catalyst/ionomer compound, which affects the homogeneity in the catalyst layer during the casting of catalyst ink, and also the electrical conductivity of the catalyst, which is the main skeleton that built the electronic channel for the electrochemical reaction, are important properties to be characterized. Figure 3a displays the pressure-dependent electrical conductivity of IrO*_x_*·*n*H_2_O and IrO_2_. The electrical conductivity increased with the elevation in the pressure for both catalysts. The electrical conductivity was 6.11 × 10^−5^ and 1.76 × 10^−3^ S/cm for IrO*_x_*·*n*H_2_O at a pressure of 1 and 12 MPa, while the value was 13.16 and 48.52 S/cm for IrO_2_. The electrical conductivity of IrO_2_ was almost four orders of magnitude higher than IrO*_x_*·*n*H_2_O, which would greatly affect the PEMWE performance in regions where ohmic polarization dominates. This result further explained the cell performance difference in these two catalysts at high current density.

High-resolution XPS spectra signals of Ir 4f for the catalyst/ionomer ink were deconvoluted into four peaks, as shown in Figure 3b. The split coupled peaks at around 61.4 and 64.5 eV belonged to Ir^0^, those at 62.0 and 65.2 eV corresponded to Ir^3+^, while the other peaks at around 63.1 and 66.4 eV contributed to Ir^4+^ [45]. A high proportion of trivalent Ir is more beneficial to OER activity according to reports in the literature [46]. The values of Ir^4+^/Ir^3+^ for IrO*_x_*·*n*H_2_O and IrO_2_ were 0.13 and 0.61, respectively. This indicated that Ir in IrO*_x_*·*n*H_2_O mainly showed a lower trivalent state and therefore possessed higher OER activity, which was consistent with the result of the electrocatalytic measurement. The Zeta potential of the catalyst ink is a key parameter to predict the stability of the homogeneously dispersed multiphase systems [47]. The Zeta potential of IrO*_x_*·*n*H_2_O and IrO_2_ were −3.50 and −2.46 mV, as shown in Figure 3c. The negative value was due to the negatively charged Ir oxide and the existence of sulfonate groups in the side chain of the ionomer [18]. The Zeta potential of ink corresponds to the interaction state between the catalyst and ionomer, especially the size of the catalyst aggregates, which greatly affects the distribution state of the ionomer. So, the particle size distribution of the catalyst ink was also investigated, as shown in Figure 3d. The distribution curve was similar, further indicating the average sizes of 1205 and 759 nm for IrO*_x_*·*n*H_2_O and IrO_2_, respectively. Typically, the formation of catalyst/ionomer aggregates is affected by both the hydrophobic interactions between the main chains and the electrostatic interactions between the side chains in the dispersion medium [13]. As mentioned above, the microstructure of IrO*_x_*·*n*H_2_O was tight aggregates composed of small nanoclusters wrapped by ionomers, which was difficult to disperse compared with IrO_2_, thus demonstrating particles with larger sizes. An ink with a large particle size usually settles quite fast, which may cause inhomogeneous of the catalyst layer [48].

In addition, HRTEM imaging and EDS scanning were also applied to clarify the interaction state of the catalyst and ionomer in the slurry ink (Figure 3e,f and Figure S2). The HRTEM technique is often used to characterize the polymer coating information of composites due to differences in crystalline properties [49,50,51]. Herein, the unique dividing lines of Ir oxides with clear lattice fringes and ionomers with amorphous structures for both samples were redrawn in detail with a yellow dotted line, as shown in Figure 3e,f. It was obvious that the amorphous region for IrO_2_ was a very thin layer (below 1 nm) uniformly covering the surface of the crystal, while the amorphous ionomer substance was unevenly distributed for IrO*_x_*·*n*H_2_O. The ionomer coating was thick at the top of the oxide aggregates but was naked without obvious coverage at the bottom of the oxide aggregates (Figure 3e). A thick ionomer will increase the resistance of mass transfer, which reduces the cell performance of PEMWE [52]. The elemental mapping of Ir, S, O, and F for IrO*_x_*·*n*H_2_O and IrO_2_ were similar (Figure S2), which uniquely demonstrated the existence of the catalyst and ionomer, confirming the uniform dispersion characteristics of catalyst inks [53]. To investigate the structure features of the catalyst layer, the cross-sectional SEM images of IrO_2_ and IrO*_x_*·*n*H_2_O in low resolution were acquired, as shown in Figure 3g,h. IrO_2_ was composed of small aggregates and displayed a uniform filling state, while IrO*_x_*·*n*H_2_O contained aggregates with various sizes, obvious ionomer aggregation, and voids. The homogeneous structure of the catalyst layer of IrO_2_, which benefitted from the good distribution of the catalyst and ionomer, played an important role in the performance of PEMWE.

SAXS is one of the most efficient methods to uncover detailed structural properties and is usually used to analyze the microphase separation of an ionomer and characterization of the aggregates [54,55]. The SAXS line profiles of IrO*_x_*·*n*H_2_O and IrO_2_ are shown in Figure 3i. The scattering vector (*q*) is inversely proportional to the particle size, and the intensity indicates the fraction of particles at that size [56]. The intensity was reduced along with the increment in *q*, while the decline in the intensity of IrO*_x_*·*n*H_2_O was larger, demonstrating the larger proportion of aggregates with a small particle size in IrO*_x_*·*n*H_2_O. This was consistent with the description of HRTEM and the size distribution of the catalyst ink above. The 2D SAXS patterns of IrO*_x_*·*n*H_2_O and IrO_2_ were similar in that an unfocused ring appeared, which indicated a good distribution of the ionomer in the catalyst layer [57]. Overall, a variety of characterization methods were adopted to evaluate the features and properties of the catalyst and corresponding ionomer-contained ink, which indicated that the anode with the IrO*_x_*·*n*H_2_O catalyst performed poorly in PEMWE due to its inferior catalyst/ionomer electrode structure and low electrical conductivity.

**Figure 4:**
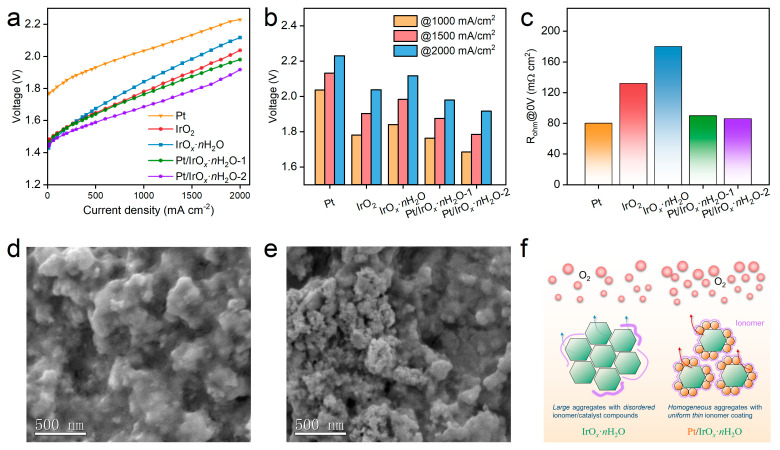
(**a**) The I-V curves of a PEMWE single cell for Pt, IrO_2_, IrO*_x_*·*n*H_2_O, Pt/IrO*_x_*·*n*H_2_O-1, and Pt/IrO*_x_*·*n*H_2_O-2; (**b**) cell voltage of Pt, IrO_2_, IrO*_x_*·*n*H_2_O, Pt/IrO*_x_*·*n*H_2_O-1, and Pt/IrO*_x_*·*n*H_2_O-2 at current densities of 1000, 1500, and 2000 mA/cm^2^; (**c**) ohmic resistance of the five samples; cross-sectional SEM images of (**d**) Pt/IrO*_x_*·*n*H_2_O-1 and (**e**) Pt/IrO*_x_*·*n*H_2_O-2; and (**f**) scheme of the newly regulated catalyst layer of Pt/IrO*_x_*·*n*H_2_O for significantly enhanced PEM water electrolysis.

Based on the analysis of anodes with the two representative commercial Ir oxide catalysts, the correlation between the differences in their PEM water electrolysis performance with the catalyst/ionomer structural characteristics, as well as the catalyst property, was basically analyzed. The commendable PEM electrolytic performance of the IrO_2_ anode stems from its nearly optimal catalytic layer structure, despite its inherent moderate activity. However, the subdued activity observed in the IrO*_x_*·*n*H_2_O anode is constrained by challenges mainly in structural homogeneity as well as conductivity. Therefore, proposing an effective approach to address it is reasonable for validating the above conclusions and achieving the purpose of performance improvement. Pt nanoparticles, a commonly used material in the hydrogen energy field with good compatibility in the catalytic slurry system of PEMWE as well as excellent electrical conductivity, were used here to regulate the homogeneity and also conductivity in the catalyst layer [58]. The newly constructed catalyst layer was assembled to MEA, and the corresponding PEMWE performance is shown in Figure 4a. The open circuit voltage of Pt-assisted MEAs were the same as IrO*_x_*·*n*H_2_O and lower than IrO_2_, indicating that the intrinsic activity of Pt was insufficient to contribute to performance. The I-V curve of Pt was also measured, which proved this and demonstrated good potential with a low rising speed of voltage. The cell performance of Pt/IrO*_x_*·*n*H_2_O-1 and Pt/IrO*_x_*·*n*H_2_O-2 were significantly improved in the range of high current density, and Pt/IrO*_x_*·*n*H_2_O-2 with a high Pt ratio performed better, demonstrating the effectiveness of the Pt component and also its ratio. The cell voltage of five different catalysts at various current densities is shown in Figure 4b. The cell voltage of the IrO*_x_*·*n*H_2_O catalyst at 1000, 1500, and 2000 mA/cm^2^ changed from 1.841, 1.964, and 2.117 V to 1.685, 1.785, and 1.917 V, demonstrating a voltage reduction of 8.5%, 9.1%, and 9.4%, respectively. In addition, the ohmic resistance of the five catalyst layers was also recorded with the high-frequency resistance (HFR) technique [59,60]. The R_ohm_ value of Pt, IrO_2_, and IrO*_x_*·*n*H_2_O were 80, 132, and 180 mΩ cm^2^. The value of Pt/IrO*_x_*·*n*H_2_O-1 and Pt/IrO*_x_*·*n*H_2_O-2 were 90 and 86 mΩ cm^2^, showing a reduction of 50% and 52%, respectively. This indicated that the reduction in ohmic resistance by Pt was limited and further revealed the new catalyst/ionomer structure was more important for the performance enhancement. Furthermore, the cross-sectional SEM images of Pt/IrO*_x_*·*n*H_2_O-1 and Pt/IrO*_x_*·*n*H_2_O-2 were recorded (Figure 4d,e). Aggregates consisting of Pt nanoparticles, IrO*_x_*·*n*H_2_O nanoparticles, and ionomers with uniform size as well as a noticeable homogeneous layer of the ionomer coating were presented in Pt/IrO*_x_*·*n*H_2_O-1. For Pt/IrO*_x_*·*n*H_2_O-2, uniform aggregates with narrower sizes and imperceptible ionomers appeared. The catalyst layer of Pt/IrO*_x_*·*n*H_2_O-2 was more reasonable with smaller aggregates and a thinner ionomer coating, which benefited from the introduction of Pt nanoparticle and thus constructed a more efficient channel for mass transfer and even electron conduction. The scheme of enhancement in the novel regulated catalyst layer consisted of evenly distributed Pt/IrO*_x_*·*n*H_2_O and ionomer, which is illustrated in Figure 4f. The introduction of the favorable Pt component optimized the aggregate behavior through its better ionomer-affinitive feature and also improved the electrical conductivity, achieving the prospect of developing high-efficiency PEM water electrolysis.

## 4. Conclusions

In this work, iridium oxide with two representative physical phases was chosen, one had a crystal lattice that was clearly ordered and loosely packed, while the other type had disordered lattice stripes and was tightly packed. Their behavior in PEMWE was tested, which was found to have a strong causal relationship with the catalyst/ionomer compound structure. Firstly, IrO_2_ was intrinsically active, while IrO*_x_*·*n*H_2_O was conductivity insufficient. Furthermore, the results of the electrocatalytic measurement and physical characterization indicated that the IrO*_x_*·*n*H_2_O anode was noticeably inhomogeneous and contained aggregates of various sizes, obvious ionomer aggregation, and voids. To improve this problematic situation, we constructed a new anode electrode with Pt-compounded IrO*_x_*·*n*H_2_O, which simultaneously optimized the aggregate’s behavior and promoted the electrical conductivity. The PEMWE performance of the IrO*_x_*·*n*H_2_O catalyst was significantly enhanced, which showed a voltage reduction of 8.5%, 9.1%, and 9.4% at 1000, 1500 and 2000 mA/cm^2^, respectively. This systematic analysis of the OER catalyst from fundamental structure to device performance and the subsequent effective performance optimization strategies are of great significance to the development and commercialization of key materials for next-generation high-performance PEM water electrolyzers.

## Data Availability

Data are contained within the article.

## Supplementary Materials

The following supporting information can be downloaded at: https://www.mdpi.com/article/10.3390/polym16020237/s1.
