# Supplementary material for: Structure–Performance Correlation Inspired Platinum-Assisted Anode with a Homogeneous Ionomer Layer for Proton Exchange Membrane Water Electrolysis
Source: Polymers (Basel). 2024 Jan 15;16(2):237. doi: 10.3390/polym16020237 (PMC10820505; doi:10.3390/polym16020237)
Supplement: Supplementary file 1 [file polymers-16-00237-s001.zip › polymers-2761628-supplementary.pdf]

Supporting information for

Structure–Performance Correlation Inspired Platinum-Assisted Anode with a Homogeneous Ionomer Layer for Proton Exchange Membrane Water Electrolysis

Feng Cheng 1,2,†, Tian Tian 1,3,†, Rui Wang 1,3,*, Hao Zhang 3, Liyan Zhu 3 and Haolin Tang 1,3,*

1 National Energy Key Laboratory for New Hydrogen-Ammonia Energy Technologies, Foshan Xianhu Laboratory, Foshan 528200, China; cf1987416@163.com (F.C.); ttcx@whut.edu.cn (T.T.)
2 Wuhan Institute of Hydrogen and Fuel Cell Industrial Technology, 555 Cultural Avenue, Hongshan District, Wuhan 430070, China
3 State Key Laboratory of Advanced Technology for Materials Synthesis and Processing, Wuhan University of Technology, Wuhan 430070, China; zhanghaoxs2023@163.com (H.Z.); z1028@whut.edu.cn (L.Z.)
* Correspondence: rwang@whut.edu.cn (R.W.), thln@whut.edu.cn (H.T.)
† These authors contributed equally to this work.

The single cell set-up was conducted as follow: The gas diffusion layer on the anode side uses platinum-coated titanium felt and titanium mesh to form a gradient diffusion structure. The current collecting plate uses a three-snake platinum-coated titanium plate. The gas diffusion layer on the cathode side is made of carbon paper (Freudenberg, E20H), and the current collecting plate is made of three-snake graphite plate. The bolt assembly pressure is 6 ~ 8 N*m. After assembly, the air tightness test is carried out. After passing the test, it is installed on the test bench for testing.

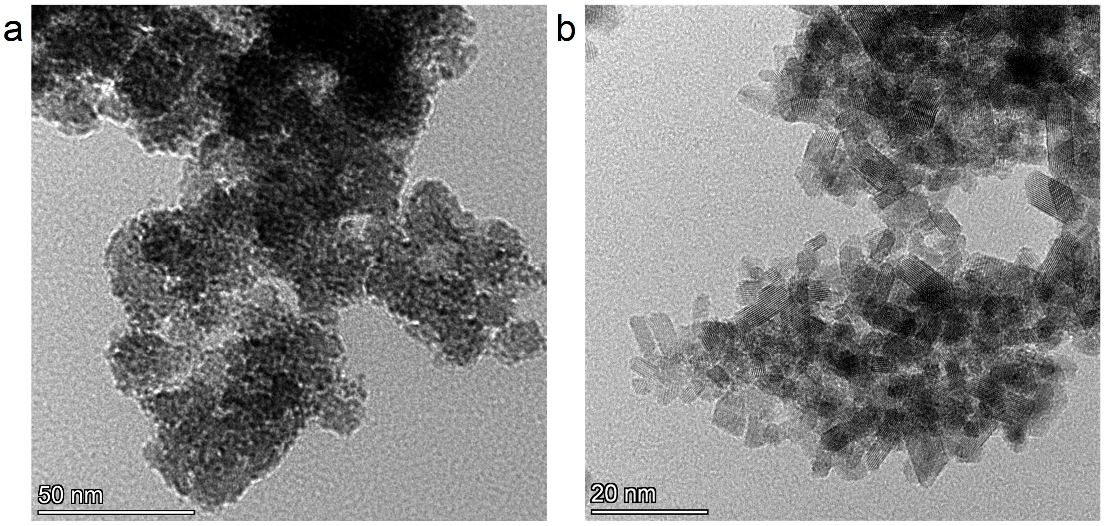

Figure s1. Low-resolution TEM images of the a) $IrO_x \cdot nH_2O$ and b) IrO_2 catalysts.

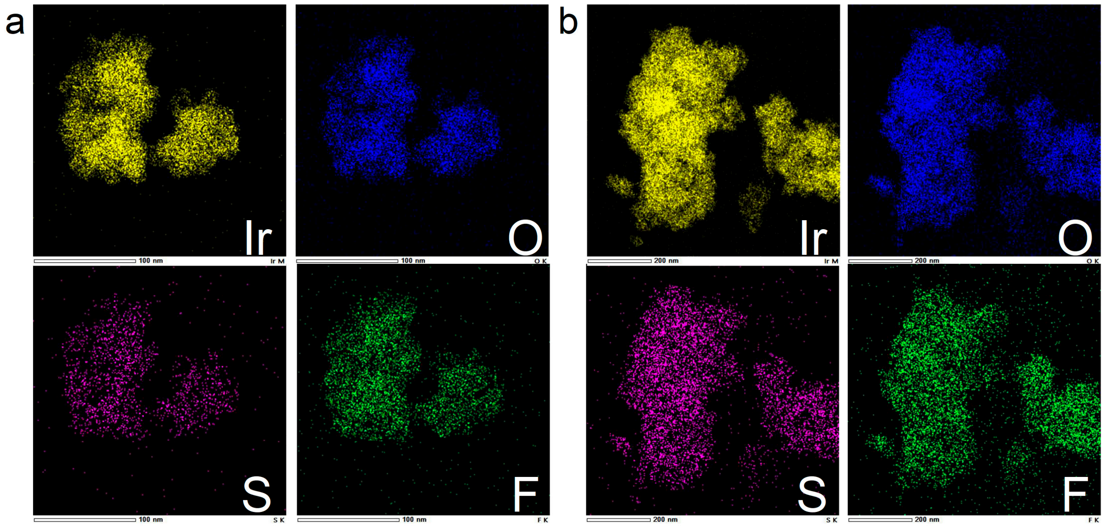

Figure s2. Elemental mapping images of element Ir, S, O, and F of a) $IrO_x \cdot nH_2O$ and b) IrO_2.

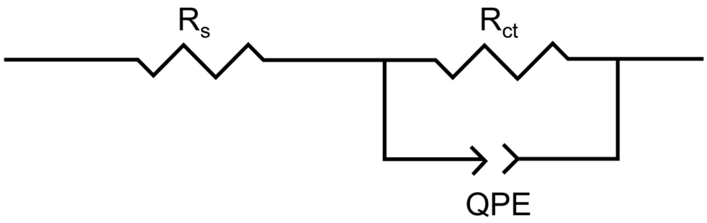

Figure s3. The equivalent circuit for the catalysts.

Table s1. Fitting results of the equivalent circuit for the catalysts

Catalyst	$IrO_x \cdot nH_2O$	IrO_2
R_s (ohm)	12.47	12.27
R_{ct} (ohm)	16.19	17.59

Table s2. The ratio of Ir (0), Ir (3^+), and Ir (4^+), for $IrO_x \cdot nH_2O$ and IrO_2

Valence	$IrO_x \cdot nH_2O$	IrO_2
Ir (0)	/	18.2%
Ir (3^+)	40%	25.4%
Ir (4^+)	60%	56.4%
